# Molecular occurrence and risk factors for *Toxoplasma gondii* infection in equids in Jilin, China

**DOI:** 10.1038/s41598-022-16658-6

**Published:** 2022-07-30

**Authors:** Wanfeng Liang, Shaowei Zhao, Nan Wang, Zeyu Tang, Fanglin Zhao, Meng Liu, Weidong Jin, Yinbiao Meng, Lijun Jia

**Affiliations:** 1 Laboratory of Veterinary Microbiology, Department of Veterinary Medicine, Agriculture College of Yanbian University, No.977 Park Road, Yanji, 133000 China; 2 Jilin Provincial Academic of Animal Husbandry and Veterinary Medicine, Changchun, 130062 China

**Keywords:** Diseases, Health care, Risk factors, Sequencing

## Abstract

*Toxoplasma gondii*, one of the important zoonotic parasites, has been detected in lots of hosts including humans, with a widespread prevalence. The products of equids, such as meat and milk, have been closely related to humans’ life. As the intermediate hosts, little is known about equids toxoplasmosis in Jilin province. Therefore, the present study aimed to evaluate the occurrence and risk factors of *Toxoplasma gondii* infections in equids from Jilin, northeastern China. In this study, a total of 245 blood samples of equids (192 horses, 25 donkeys and 28 mules) were collected from six localities in Jilin Province from March 2018 to August 2020 and detected by PCR. The occurrence rate of *T. gondii* B1 gene was analyzed using multivariable logistic regression to evaluate risk factors associated with the positive rates in equids. Among 245equids, *T. gondii* molecular occurrence was 9.0% (22/245). The highest positive rate was observed in equids from Dongfeng (16.3%) followed by Taonan (10.0%), Wangqing (8.3%), Antu (8.0%), Tonghua (8.0%) and Shulan (2.3%). Statistical analysis revealed that farming model and region may be two main risk factors. Data analysis indicated that the positive rate in captive farm (3.2%, 95% CI: 0.0–6.7%) was significantly lower than those in cage-free farm (P < 0.05), and the region of Shulan was protective factor (OR: 0.063, 95% CI: 0.007–0.559).The results of our study alert people to be aware that the present of equids *T. gondii* infection in this region, and contribute to a prevention and treatment program for toxoplasmosis in Jilin, China.

## Introduction

*Toxoplasma gondii* (*T. gondii*) is an obligate intracellular pathogen which can cause infection in almost all warm-blooded animals, including humans^1^. Felids are the only known definitive hosts, which play a key role in the spread of diseases by excreting oocysts of *T. gondii* in their faeces. As a common courtyard animal, domestic cat is a considerable risk factor for the infection of *T. gondii*. One cat may shed millions of oocysts within a week during primary infection^2,3^. In humans or other intermediate hosts, the routes of infection with *T. gondii* include ingestion of water or food contaminated with oocysts shed by felids, ingestion of meat containing active tissue bradyzoites, and vertical transmission during gestation^4^. The infection of toxoplasmosis in different animals can have various clinical symptoms. Host animals infected with toxoplasmosis may appear to be fever, lymphadenopathy, ocular inflammation and central nervous system disturbances etc. Normally, the infections in humans are asymptomatic, but at times the parasite can have serious consequences. For example, toxoplasmosis can cause abortion in primary infected pregnant women; in addition, this disease can be fatal for immunocompromised individuals^5,6^.

Since it was first isolated in 1908, *T. gondii* has a cosmopolitan distribution. Serosurvey has been carried out in sheep and goats in Northern Iraq, with a seroprevalence of toxoplasma infection of 42.1% and 36.1%^7^. Using the Sabin–Feldman dye test, the seroprevalence in wild sika deer surveyed in Japan was 47.5%^8^. The prevalence of *T. gondii* infection in dogs has been investigated in Brazil, which detected a seroprevalence of 9.54% in samples^9^. In Nordic-Baltic region, the positive rates of *T. gondii* infection in five animal species were statistically analyzed, which draw a conclusion that the seroprevalence of *T. gondii* in animals ranged from 6 to 33%^10^. Equids infected with *T. gondii* were also reported in the previous studies. Rodrigues et al. found that the seroprevalence of *T. gondii* in donkeys from Portugal was 5.9%^11^. In Ukraine, the horses infected with *T. gondii* was estimated of 21.1%, using the method of enzyme-linked immunosorbent assay (ELISA)^12^.

China is located in the world's highest level of the breeding stock of donkeys and horses, with world rankings of second and third, respectively^13^. The areas of northeast, north, and north-west China are the primarily breeding bases of equids. Recently, the horse and donkey industries have been related to many public industries, such as tourism, entertainment, competition, food industries, and pharmaceutical industries, which are closely related to people's health. Jilin Province is located in the northeast China. The prevalence of *T. gondii* in animals was occasionally reported in Jilin, such as wild waterfowls 7.2%, pig 7.8%, and chicken 8.9%^14–16^. Although the infection with *T. gondii* in animals has been verified a potential threat for people's health in Jilin province, there are few studies involving the molecular occurrence of *T. gondii* infection in equids. Thus, this study aimed to determine the occurrence of *T. gondii* in equids from Jilin, northeastern China. Meanwhile, we also sought to determine the potential risk factors of infection with *T. gondii* in equine among different conditions (for example region, sex and management, et al.).

## Materials and methods

### Ethical statement

The samples collected in this study was permitted by the farm owners, and equids involved in sampling were well looked after. All experimental procedures in animals were conducted in strict following the Animal Ethics Procedures and Guidelines of the People's Republic of China and Ethical Principles in Animal Research issued by Yanbian University, and approved by the Animal Ethics Committee of Yanbian University. This study was carried out in compliance with the ARRIVE guidelines.

### Sample collection

**Figure 1:**
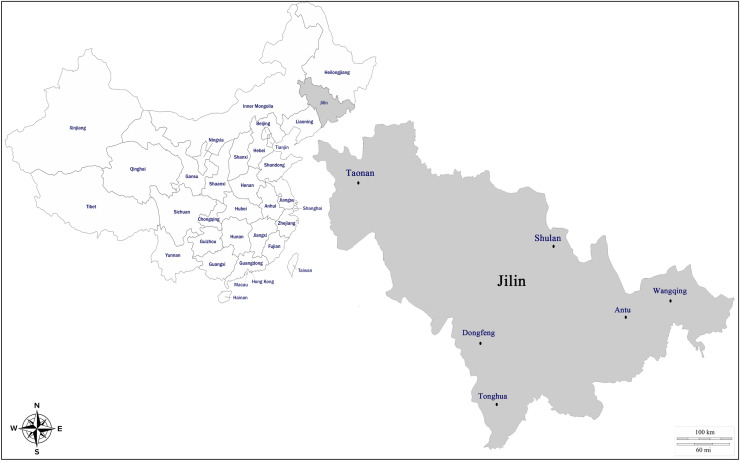
Map of the sampling districts in Jilin, northeastern China. The map was generated by DataV.GeoAtlas (http://datav.aliyun.com/portal/school/atlas/area_selector) and Adobe Illustrator software (2021). All locations sampled in this stud-y were marked in the enlarged map of Jilin Province.

In total, 245 blood samples of equids, including 192 horses, 25 donkeys and 28 mules, were collected from the following six counties in Jilin Province between March 2018 and August 2020: Dongfeng; Taonan; Shulan; Wangqing; Antu and Tonghua (Fig. 1). All samples were aseptically collected from the jugular vein of animals using K2-EDTA vacuum tubes (Solarbio, Beijing, China) .Blood samples were then carefully stored at − 20 °C until DNA extraction. A predesigned questionnaire consisting of information, such as region, sex and management, of the animals was filled out with the help of farm owners to evaluate the risk factors associated with occurrence of *T. gondii*.

### DNA extraction and PCR amplification

The blood extraction kit (Vazyme, Nanjing, China) was used to extract the genomic DNA from blood samples of the investigated equids, according to the manufacturer's instructions. The genomic DNA was aliquoted and stored at − 20 °C until conventional PCR tests were performed. The PCR amplification, using *T. gondii*-specific primers to target the B1 gene, was conducted in a 25 μl reaction mixture comprised of 0.5 μl of each primer, 2 μl template DNA, 1 μl dNTP mix (TaKaRa, Dalian, China), 2.5 μl of 10 × Ex Taq buffer, 0.25 μl Ex Taq (TaKaRa), and 18.25 μl distilled water^17^. Genomic DNA extracted from the RH strain of *T. gondii* was used as a positive control. The negative control was distilled water. The information of primers and procedure of PCR was presented in Table 1.

**Table 1:** Primer sequences for the amplification of *T. gondii*.

Organisms	Target genes	Assay	Amplicon size (bp)	Primers	Sequence 5′–3′	Annealing temperature (°C)	Reference
*T. gondii*	B1	PCR	194	F	GGAACTGCATCCGTTCATGAG	57 °C	^17^
				R	TCTTTAAAGCGTTCGTGGTC		

### Sequencing and phylogenetic analysis

The Gel Extraction Kit (OMEGA, Norcross, GA, USA) was used to purify the amplicons from B1 gene-positive samples. The purified amplicons was cloned into the PMD 18T-Simple Vector (TaKaRa), which was further transformed into the competent DH5α cells (TaKaRa). After precisely identified, the positive amplicons was sequenced by Shanghai Yingweijieji Biotechnology Company.

**Figure 2:**
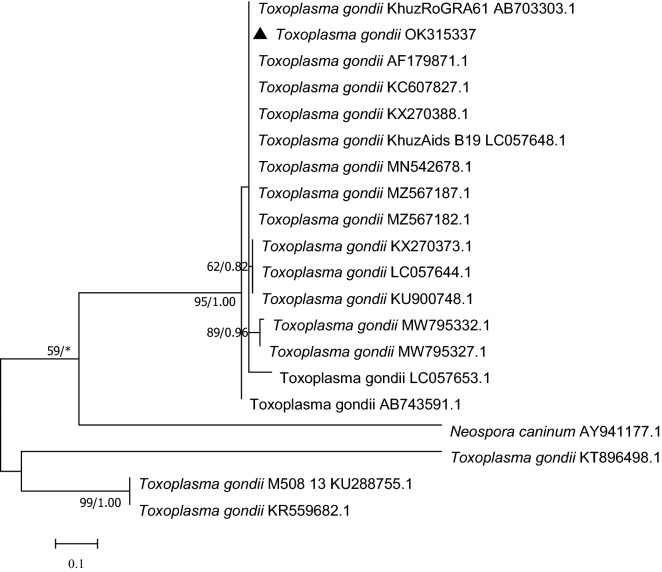
Phylogenetic tree based on the B1 gene of *T. gondii* isolates from naturally infected equids and those previously deposited in the GenBank database. The ML tree and Bayesian inference were implemented by MEGA7 with a Kimura-2-parameter model and MrBayes3.2 with the HKY + I model, respectively. ML Bootstrap values < 50 and BI posterior probabilities < 0.80 were not shown. The gene sequence marked with triangle was obtained in this study.

The BLASTn program on the NCBI website was used to a BLAST analysis of sequences obtained in this study, and relative sequences were further aligned using ClustalW before manually edited by Bioedit v.7.0.9 software (Fig. 2)^18^. The methods of maximum likelihood (ML) (MEGA v.7.0) and Bayesian (MrBayes 3.2) were executed for phylogenetic analysis^19^. In the ML analysis, the substitution model Kimura-2-parameter accompanying with 1000 replicates of bootstrapping was performed using MEGA 7.0. For Bayesian analysis, four Monte Carlo Markov chains for 10^6^ generations with sampling every 10^3^ generations was performed using the HKY + I model by MrBayes 3.2 software. The sampled trees of initial 25% were discarded as ‘burn-in’.

### Statistical analysis

The variables associated with the *T. gondii* infection, for example region, sex and management, were analyzed using the current data by Fisher’s exact or Chi-square test. All variables with P ≤ 0.05 in the bivariate analysis were selected for analysis with a stepwise backward logistic regression analysis^20^. The issue of multicollinearity among variables was assessed by variance inflation factor (VIF).The Hosmer–Lemeshow test, Negelkerke R2 test, and the observed versus predicted values (residual statistics) were calculated to assess the fitness of the final models^21^. The odds ratios (ORs) and their 95% confidence intervals (95% CIs) were also computed. The data was analyzed with SPSS software version 19.0 (IBM SPSS Statistics for Windows, Version 19.0. Armonk, IBM Corp., NY, USA), and a P < 0.05 was considered statistically significant.

## Results

### Molecular occurrence of *T. gondii*

In the present study, DNA of *T. gondii* was detected in 22 of the blood samples of equids indicating an overall positive rate of 9.0% (22/245) infected with *T. gondii*. Among the six regions tested for *T. gondii* infection, occurrence rate was highest in county of Dongfeng (16.3%, 95% CI: 5.6–27.1%), followed by Taonan (10.0%, 95% CI: 0.0–21.4%), Wangqing (8.3%, 95% CI: 1.8–14.9%), Antu (8.0%, 95% CI: 0.0–19.4%), Tonghua (8.0%, 95% CI: 0.0–19.4%) and Shulan (2.3%, 95% CI: 0.0–6.9%). Compared with Shulan (2.3%, 1/44), the infection rate of *T. gondii* in Dongfeng is in the higher level (16.3%, 8/49), and the occurrence rate among these two regions differed significantly (P ≤ 0.05) (Table 2). In the preliminary evaluation, another three categories, including management, sex and species, were also analyzed for risk factors of *T. gondii* infection using the univariate model. Amplicons of *T. gondii* were detected in 19 (12.7%, 95% CI: 7.3–18.1%) of 150 equids from cage-free farms, which was significantly higher than the occurrence detected in the captive farms (3.2%, 95% CI: 0.0–6.7%). The occurrence between different species ranged from 8.0% of donkeys to 10.7% of horses, but the difference was not statistically significant (P > 0.05).Compared with the males (7.7%), the female equids (9.6%) was more prone infection with *T. gondii*. The variable of gender results no significant for the occurrence of *T. gondii* infection in this study (P > 0.05) (Table 2).

**Table 2:** Univariate analysis of risk factors associated with *Toxoplasma gondii* infection in equids in Jilin, China.

Variable	Samples tested (positive/total samples tested)	Prevalence (%)	95% CI	OR	95% CI	*P*-value
**Region**						
Dongfeng	8/49	16.3	5.6–27.1	Ref		
Taonan	3/30	10.0	0.0–21.4	0.57	0.14–2.34	0.65
Wangqing	6/72	8.3	1.8–14.9	0.47	0.15–1.44	0.18
Antu	2/25	8.0	0.0–19.4	0.45	0.09–2.28	0.53
Tonghua	2/25	8.0	0.0–19.4	0.45	0.09–2.28	0.53
Shulan	1/44	2.3	0.0–6.9	0.12	0.01–1.00	0.05*
**Management**						
Cage-free	19/150	12.7	7.3–18.1	Ref		
Captive	3/95	3.2	0.0–6.7	0.23	0.07–0.78	0.011*
**Species**						
Mule	3/28	10.7	0.0–22.9	Ref		
Donkey	2/25	8.0	0.0–19.4	0.73	0.11–4.73	1.00
Horse	17/192	8.9	4.8–12.9	0.81	0.22–2.96	1.00
**Sex**						
Male	6/78	7.7	1.6–13.7	Ref		
Female	16/167	9.6	5.1–14.1	1.27	0.48–3.39	0.63
Total	22/245	9.0	5.4–12.6			

Ref, reference; 95% CI, confidence interval; *P < 0.05, **P < 0.01.

In the multivariate logistic regression analysis, VIF analysis was executed for detecting multicollinearity among all variables. The result of VIF < 1.13 meant there was no multicollinearity. A Hosmer–Lemeshow test (χ^2^ = 7.274, df = 8, P = 0.507) and Nagelkerke R^2^ (0.17) values suggested that this model was a good fit. The results of the multivariate logistic analysis showed that the factors, which were significantly associated with the occurrence of *T. gondii* infection*,* were captive (P < 0.005, OR: 0.118, 95% CI: 0.031–0.453) and the region of Shulan (P < 0.05, OR: 0.063, 95% CI: 0.007–0.559). Equids in the two factors were significantly less likely to be positive of *T. gondii* (Table 3).

**Table 3:** Results of the multivariable logistic regression analysis for the prevalence of *Toxoplasma gondii*.

Variable	Category	P-value	Odds ratio	95% CI
Management	Cage-free	a	0.118	0.031–0.453
	Captive	0.002**		
Region	All other regions	a	0.063	0.007–0.559
	Shulan	0.013*		

a, baseline; 95% CI, confidence interval.

### Sequencing and phylogenetic analysis

In this study, the B1 gene of *T. gondii* was identified by the method of PCR. The amplicons was successfully obtained and sequenced for nucleotide analysis. Nucleotide sequence identity data demonstrated that the *T. gondii* B1 gene sequence (GenBank: OK315337) obtained in this study shares 99.5% sequence identity with Mexico (GenBank: KX270373) and was 100% identical to the sequence of India (GenBank: KC607827). For phylogenetic analysis, the phylogenetic tree of *T. gondii* was constructed using the B1gene sequences obtained in our study and those deposited in the GenBank database. A single tree topology converged with ML and BI analyses was presented with support values (ML/BI). The phylogenetic tree showed evidence of two main clades and indicated that the *T. gondii* B1 gene amplified in this study formed one cluster with the isolates from Iran (GenBank: KC607827), Mexico (GenBank: KX270388) and Iraq (GenBank: MZ567182) et al., with a range of 89.1–100% nucleotide homology (Fig. 2).

## Discussion

According to the published reports, more than 350 host species can be infected with *T. gondii*^22^. Toxoplasmosis, caused by the causative agent of protozoan *T. gondii*, has been a significant threaten to public health in the world. There is nearly one third of the human population estimated to be infected with *T. gondii*^23^. The equids, such as horse and donkey, was a kind of livestock with a long history. Nowadays, their products, including milk and meat, were also closely related to humans’ life. In this study, we found that the overall molecular occurrence of *T. gondii* in equids in Jilin was 9.0%, which was higher than those in horses (5.15%) and donkeys (6.48%) detected in the previously studies^24,25^. This discrepancy in the positive rates of *T. gondii* in equids among studies may be related to feeding purposes, sampling positions and detection methods, etc. In these previous studies, the samples of horses/donkeys tested for infection with *T. gondii* was totally collected from slaughter houses, which meant the horses/donkeys was raised for food sources. However, equids collected in this study were raised for farm working or leisure activities besides food sources. Compared with slaughtered equids, other equids lived in a looser livelihood conditions, increasing the risks of infection with *T. gondii*. In addition, *T. gondii* can invade into multiple organs, including lungs, liver, and brain, of hosts via blood or lymph systems^5^. The differences of sampling positions may also lead to disparity in *Toxoplasma* infections among studies.

In the statistical analysis, we found an association between *T. gondii* infection and farming mode or living in Shulan. It was observed that the equids raised in “Cage-free” farms had a significantly higher positive rate (12.7%) compared to “Captive” farms (3.2%). Previously studies showed that undetected environmental oocyst transmission is one of the major routes of *T. gondii* transmission^2,26,27^. Equids fed in the “Cage-free” farms have a high risk of being exposed to the environments contaminated by sporulated oocysts, such as contaminated food/soil/water with oocysts, domestic and feral cats infected with *T. gondii*, which may increase the risks of *T. gondii* infection. Compared with other regions, the samples collected from Shulan had the lowest positive rate (2.3%). This discrepancy of *T. gondii* occurrence in equids among different geographical regions may be related to the socio-economic status and regional climate. The environment with relative high temperature and humidity may increase the viability of the oocysts, which may lead a higher infection of *T. gondii*^6,28^. However, Shulan is located in the north-central part of Jilin Province, with the lower temperature that may be inclement for the survival of oocysts shed by the definitive hosts. Among these regions sampled in this study, Shulan has a higher socio-economic status. The districts with high-income levels has access to better sanitation facilities and safe food/water, providing good hygienic conditions for intermediate hosts, which would reduce the chance of contacting with oocysts. The molecular occurrence of 10.7% found in the mules was higher, compared with the occurrence detected in horses (8.9%) and donkeys (8.0%) in current study. The most of mules sampled in this survey were raised in the backyards, with the surroundings of cats occasionally haunting, which may be a crucial factor for increasing the infection with *T. gondii* in mules. Tachyzoites, one of the infective forms of *T. gondii*, can invade the fetus via transplacental migration, causing fetal abnormalities, neonatal death or abortion of pregnant hosts^29,30^. Although, there were no significant difference in the positive rates of *T. gondii* between male (7.7%) and female (9.6%) equids found in this study, the condition of occurrence in female remains a need to strengthen supervision.

*Toxoplasma gondii*, one of the most important foodborne pathogen, was once deemed as one of the three pathogens, including Salmonella and Listeria, which together account for > 75% of all deaths caused by foodborne disease in the USA^31^. The prevalence of toxoplasmosis in equids had ever been reported in few provinces of China, for example Shandong (17%), Yunnan (27.1%)^32,33^. Primary infection with *T. gondii* is usually asymptomatic or moderate symptoms. Therefore, grasping the present situation of toxoplasmosis in animals, which is closely related to humans, can significantly reduce threats to public health. In this study, B1 gene, one of the multi-copy sequence specific and highly conserved gene of *T. gondii* strains, was amplified for detecting the occurrence in equids, and an overall positive rate of 9.0% was found in samples^34^. Although, there may present varying degrees of difference among surveys in the prevalence of *T. gondii* due to the different of laboratory tests or sample size. Our finding that regions sampled in this survey were 100% positive of *T. gondii* infection.

The results of present study showed that equids in Jilin was infected with varying degrees of *T. gondii*. Therefore, equids farmers in these regions should enhance the awareness of prevention and control of toxoplasmosis. Cats, which were commonly found in the backyards, play a vital role in contaminating the environment with oocysts, because the sexual reproduction may be reactivated to complete the life cycle of *T. gondii*, when the tissues of intermediate hosts was consumed by cats^35^. Therefore, the deratization carried out in farm is also significantly important for reducing the occurrence of *T. gondii* in equids.

## Conclusions

The present study revealed a 9.0% molecular occurrence of *T. gondii* infection in equids in Jilin province. Farming mode was likely to be a primary risk factor for *T. gondii* infection in equids of the northeastern region in China. Taken with other investigations, the results of our study provide baseline data for the prevention and control of toxoplasmosis in this region, and alert people to be aware that the conditions of animals infected with *T. gondii* was commonly found in Jilin, China.

## Data Availability

All data supporting the conclusions of this article are included within the article.

## Abbreviations

*T. gondii*: Toxoplasma gondii
PCR: Polymerase chain reaction tests
